# Purification of Extracellular Protease from *Staphylococcus simulans* QB7and Its Ability in Generating Antioxidant and Anti-inflammatory Peptides from Meat Proteins

**DOI:** 10.3390/nu15010065

**Published:** 2022-12-23

**Authors:** Hongying Li, Hongbing Fan, Kuan Lu, Qiujin Zhu, Jianping Wu

**Affiliations:** 1 Laboratory of Animal Genetics, Breeding and Reproduction in the Plateau Mountainous Region, Ministry of Education, College of Life Sciences, Guizhou University, Guiyang 550025, China; 2 Department of Agricultural, Food and Nutritional Science, University of Alberta, 4-10 Ag/For Building, Edmonton, AB T6G 2P5, Canada; 3 School of Liquor and Food Engineering, Guizhou University, Guiyang 550025, China

**Keywords:** microbial extracellular protease, inflammation, superoxide

## Abstract

Proteases, especially microbial proteases, are widely used in food processing. The purpose of this study was aimed to purify an extracellular protease produced by the strain *Staphylococcus simulans* QB7 and to evaluate its ability in hydrolyzing meat proteins and generating antioxidant and anti-inflammatory peptides. The optimal conditions for producing the enzyme were as follows: inoculum ratio, 10%; initial pH, 6.5; temperature, 32 °C; incubation time, 36 h; and rotation speed, 160 rpm. The protease had a molecular weight of approximately 47 kDa, possessing the optimal activity at 50 °C, pH 7.0, The protease was stable at pH 4.0–8.0 and 30–60 °C, and the activity was improved by Na^+^, Mg^2+^, Ca^2+^, and Zn^2+^ ions, whereas it was inhibited by Cu^2+^, Co^2+^, Fe^3+^, Ba^2+^, Fe^2+^, β-M, and ethylene diamine tetraacetic acid disodium salt (EDTA). The protease could effectively hydrolyze meat proteins, and the generated hydrolysate could significantly inhibit tumor necrosis factor-alpha (TNFα)-induced oxidative stress, including superoxide and malondialdehyde levels and inflammation (vascular adhesion molecule-1 [VCAM-1] and cyclooxygenase 2 [COX2)) in human vascular EA.hy926 cells. The present findings support the ability of *S. simulans* QB7 protease in generating antioxidant and anti-inflammatory peptides during the fermentation of meat products.

## 1. Introduction

Proteases are widely used in food processing (i.e., dairy, bakery, meat), pharmaceutical manufacturing, peptide synthesis, and wastewater treatment [1,2]. Proteases can be derived from three sources: animals, plants, and microorganisms [3]. Among them, microbial proteases are preferred over their plant and animal counterparts due to the relatively short growth cycle of microorganisms and scalability for industry application [4]. In recent years, the development and use of microbial proteases in the industry have been receiving increasing attention and account for approximately 60% of global industrial enzymes revenue [5]. To date, a large array of intracellular and/or extracellular microbial proteases are produced from *Bacillus*, *Alteromonas*, *Pseudomonas*, *Shewanella*, *Yersinia*, *Flavobacterium*, coagulase-negative staphylococci (CNS), lactic acid bacteria (LAB), and among others [6,7,8,9].

CNS is the dominant microorganism in fermented meat; it has a complex enzymatic system consisting of a wide variety of enzymes that hydrolyze proteins, carbohydrates, and lipids [10]. Previous studies have suggested that CNS produces proteases that degrade meat proteins into peptides and amino acids, which contribute to the characteristic flavor of fermented meat products [11]. Several strains of CNS, such as *Staphylococcus epidermidis*, *Staphylococcus xylosus*, and *Staphylococcus carnosus*, were isolated from Harbin dry sausages; extracellular proteases produced by these strains were responsible for the hydrolysis of myofibrillar proteins into smaller peptides in Harbin dry sausages [12,13]. A *S*. *simulans* strain with the ability to secret high proteolytic activity identified from Chinese dry sausages was able to degrade meat proteins into smaller peptides with in vitro antioxidant and antihypertensive activities [14,15,16].

Although several studies have reported that *S*. *simulans* strain secreted proteases in fermented meat products, purification and characterization of these proteases from *S. simulans* strain are rare. Moreover, there is limited knowledge on the role of proteases produced by *S*. *simulans* in protein degradation and their capacities in generating bioactive peptides. Our group previously isolated a CNS strain, *S*. *simulans* QB7, from *Qianwufu* sausage (a Chinese dry fermented sausage), showing high proteolytic activity in degrading pork meat proteins [17]. Thus, it is interesting to purify the major protease produced by this strain and clarify its contribution in generating bioactive peptides. In the present study, the extracellular protease was first purified using 80% ammonium sulfate precipitation, anion exchange chromatography, and size-exclusion chromatography, followed by exploring the enzymatic properties of the purified protease. Moreover, the capacity of the purified extracellular protease in degrading pork meat proteins into smaller peptides and the antioxidant and anti-inflammatory activities of the generated peptides were evaluated.

## 2. Materials and Methods

### 2.1. Reagents

DEAE Sepharose Fast Flow, Sephadex G-75, and Coomassie brilliant blue G-250 were purchased from Solabio Technology Co. Ltd. (Beijing, China). Trichloroacetic acid (TCA), ethylenediaminetetraacetic acid disodium salt (EDTA), β-mercaptoethanol (β-M), and ammoniummum sulfate were purchased from Sigma-Aldrich (St. Louis, MO, USA). Human vascular endothelial cells of the type EA.hy926 (CRL-2922) were purchased from American Type Culture Collection (Manassas, VA, USA). Dulbecco’s modified Eagle’s medium (DMEM), nonessential amino acids (NEAA), penicillin-streptomycin (pen-strep), fetal bovine serum (FBS), 4-(2-68 hydroxyethyl)-1-piperazineethanesulfonic acid (HEPES), and 0.25% (*w*/*v*) trypsin-0.53 mM EDTA were purchased from Gibco Invitrogen (Burlington, ON, Canada). Dithiothreitol (DTT) and Triton-X-100 were obtained from Sigma-Aldrich (St. Louis, MO, USA). Tumor necrosis factor-alpha (TNFα) was obtained from R&D Systems (Minneapolis, MN, USA). Dihydroethidium (DHE) was purchased from Biotium (Fremont, CA, USA).

### 2.2. Optimization of Culture Conditions for Protease Production

*S. simulans* QB7 was previously isolated, identified by whole-genome sequencing (WGS) (the accession number PRJNA854621), and stored in the China Center of Industrial Culture Collection. *S. simulans* QB7 was activated in mannitol salt broth (MSA) (beef extract, 5.0 g/L; peptone, 10.0 g/L; D-mannitol, 10 g/L; sodium chloride, 75 g/L; pH, 7.2 ± 0.2). The initial culture conditions for protease production were optimized in mannitol salt agar (MSA), including inoculum ratio (2%–16%), initial pH (3.5–9.0), incubation temperature (17–52 °C), incubation time (0–96 h), and rotation speed (100–180 rpm). Three independent experiments were conducted.

### 2.3. Protease Activity Assay

The protease activity was evaluated according to a previously proposed method [13] with minor modifications. An aliquot (0.5 mL) of protease solution was mixed with 0.5 mL of 2% casein solution in 0.02 M phosphate buffer at pH 7.0 and incubated in water bath for 10 min at 40 °C. Subsequently, the enzymatic reaction was terminated by the addition of 1 mL of 0.4 M TCA. The mixture was placed in an ice bath for 10 min and then centrifugation for 15 min at 5000× *g*. Subsequently, 0.5 mL of the supernatant was mixed with 2.5 mL of Na_2_CO_3_ (0.4 M) and 0.5 mL of Folin reagent in a 10-mL test tube; the mixture was incubated in a water bath at 40 °C for 20 min and then cooled on ice. The absorbance of the solution was determined at 680 nm in a microplate reader (Thermo Fisher Scientific, Shanghai, China). The control group was prepared by adding TCA solution prior to adding the protease solution. One unit of protease activity was defined as the amount of enzyme that released 1 µg of tyrosine per min and was expressed in U/mL, which represented the ratio of protease activity to the volume of protease solution. Protein concentration was determined using the BCA assay with bovine serum albumin (BSA) as a standard. Each measurement was repeated three times.

### 2.4. Purification of S. simulans QB7 Extracellular Protease

Crude protease extract was prepared based on the method proposed by Farhadian, Asoodeh, and Lagzian [18]. Fermented broth (1000 mL) was centrifuged at 10,000× *g* for 15 min at 4 °C, and the supernatant was considered the crude extracellular protease extract and was stored at 4 °C for further experiments.

The protease was purified according to a previously proposed method [19], with minor modifications. The supernatant of the crude protease extract was precipitated using 80% of ammonium sulfate and overnight storage at 4 °C, followed by centrifugation for 15 min (10,000× *g*, 4 °C). Subsequently, the precipitate was dissolved in Tris-HCl buffer (pH 9.0) and then dialyzed for 24 h in Tris-HCl buffer (pH 9.0). The protease solution after dialysis was loaded on the DEAE-Sepharose™ FF column (21 mm diameter × 150 mm) and purified according to a method proposed elsewhere [20]. The DEAE-Sepharose™ FF column was equilibrated with 0.05 M Tris-HCl buffer (pH 9.0). The protease solution was eluted at a flow rate of 1 mL/min with 0 to 1 M NaCl in 0.05 M Tris-HCl buffer (pH 9.0), and fractions were collected at 10-mL intervals, and then protease activity was measured. The fractions with the highest protease activity were collected and loaded into a Sephadex G-75 column (30 mm diameter × 1000 mm), which was equilibrated and eluted with 0.1 M NaCl in Tris-HCl buffer (pH 9.0) at a flow rate of 1 mL/min. Fractions with the highest protease activity were collected and freeze-dried, then stored at −20 °C until further analysis.

### 2.5. Sodium Dodecyl Sulfate-Polyacrylamide Gel Electrophoresis (SDS-PAGE)

Molecular weight and purity of *S. simulans* QB7 extracellular protease at different purification stages were determined by SDS-PAGE according to the Laemmli method Laemmli [21]. A protein marker mixture (Takara Biotechnology, Dalian, China) was applied to calibrate the molecular weight of purified protease. An aliquot of the sample (20 μL) was added to a 5% stacking gel and 12% resolving gel, and gels were stained in 0.25% Coomassie blue (R-250) solution for 0.5 h at 25 °C under a rotation speed of 100× *g* and then de-stained in 10% acetic acid solution for 2 h.

### 2.6. Enzymatic Properties

#### 2.6.1. pH and Temperature

The effects of pH and temperature on *S. simulans* QB7 protease activity and stability were determined according to the method proposed by Wang et al. [13]. Protease solutions at different pH, i.e., within pH values 3–5 using 0.1 M of lactic acid buffer, were employed; pH values were within 6–8 using 20 mM phosphate buffer; and pH values were within 9–11 using 0.1 M Borax-NaOH buffer, and the proteases were incubated at 37 °C for different incubation times (i.e., 0, 20, 40, 60, 80, and 100 min). Protease activity was measured using 2% (*w*/*v*) casein as a substrate at 40 °C for 10 min to determine the optimum pH for enzymatic activity.

In addition, after determining the optimum pH for protease activity, the effect of temperature (20–80 °C) on protease activity was investigated in protease solution at optimum pH. Protease solutions were incubated for 0, 20, 40, 60, 80, and 100 min under different temperatures, and protease activity was subsequently measured according to 2.3. The results were expressed as the relative activities based on the highest activity (considered as 100% of activity). Each measurement was repeated three times independently.

#### 2.6.2. Metal Ions and Inhibitors

The effect of different metal ions and inhibitors on protease activity was investigated under optimum pH and temperature for protease activity based on the method of Wang et al. [22]. The experiments were conducted three times independently. Protease solutions were dissolved in 5 mmol/L and 10 mmol/L solutions of different metal ions (i.e., Na^+^, Mg^2+^, Ca^2+^, Zn^2+^, Cu^2+^, Co^2+^, Fe^2+^, Ba^2+^, and Fe^3+^) and inhibitors (EDTA and β-M), and protease activity was determined as detailed 2.3. Protease activity in solution with no added metal ions or inhibitors was considered as 100% of activity (control sample).

### 2.7. Degradation of Meat Proteins by S. simulans QB7 Extracellular Protease

Meat protein was extracted from pork tenderloin according to the method [23]. Meat protein was hydrolyzed according to the method [24]. Briefly, the protein isolate (approximately 94% protein) was dissolved in ddH_2_O (5%, *w*/*w*). After heating at 90 °C for 10 min for protein denaturation, the slurry was hydrolyzed by *S. simulans* QB7 protease in a jacket beaker connected with a Titrando (Metrohm, Herisan, Switzerland) and a circulating water bath (Brinkman, Mississauga, ON, Canada) under constant pH and temperature, respectively. Protein hydrolysates were prepared using protease (4% enzyme/substrate, E/S, *w*/*w*) for 5 h at 50 °C at pH 7.0. After hydrolysis, the slurry was heated at 95 °C for 10 min to terminate the reaction and then submitted to centrifugation at 10,000× *g* for 15 min at 4 °C. The supernatant was freeze-dried and kept at −20 °C for further analysis. The hydrolysate was obtained in triplicate.

### 2.8. Size Exclusion Chromatography

The distribution of the molecular weight of hydrolysate was analyzed according to a method proposed elsewhere [24] by size exclusion chromatography using a Superdex peptide 10/300 GL column at room temperature connected to an AKTA explorer 10XT system (GE Healthcare, Uppsala, Sweden). Samples were dissolved in 30% ACN (dissolved in ddH_2_O, *v*/*v*) containing 0.1% TFA. Samples (100 µL, 1 mg/mL) were injected into the column and eluted using an isocratic gradient at a flow rate of 0.5 mL/min, and peaks were detected at 220 nm. Cytochrome C, aprotinin, vitamin B12, and (glycine) _3_ were used as molecular weight markers.

### 2.9. Cytotoxicity, Oxidant Stress, and Anti-Inflammatory Evaluation through EA. Hy926 Cells

#### 2.9.1. Cell Culture of EA.hy926 Cells

EA.hy926 cells (from 6–10 passages) were cultured based on the procedures described elsewhere [25]. Cells were grown in complete media (DMEM supplemented with 10% FBS, 25 mM HEPES, 1% nonessential amino acids, and 1% penicillin-streptomycin) in a 75-cm^2^ flask at 37 °C under 100% humidity and 5% CO_2_; the medium was changed every three days. For all experiments conducted, cells were first seeded onto 48-well plates, grown a in complete medium, and then replaced with quiescing media (same as a complete medium but with 1% FBS) for various treatments, except for the cytotoxicity test.

#### 2.9.2. Desalting Protocol, Cytotoxicity, Lipid Peroxidation, and Superoxide Detection

Prior to treatment with cells, protease hydrolysate was desalted according to the protocol described by Fan et al. [26]. Lipid peroxidation and superoxide generation in EA.hy926 cells were determined using the assay as suggested elsewhere [27].

#### 2.9.3. Anti-Inflammatory Assay in EA.hy926 Cells

The effect of the treatment with protease hydrolysates on the generation of inflammatory proteins was evaluated after 6 h of TNFα induction EA.hy926 cells. Cells were treated with 2.5 mg/mL of protease hydrolysate for 24 h, and then 10 ng/mL of TNFα was added to the solution for co-treatment for 6 h, and the production of vascular cell adhesion molecule-1 (VCAM-1) and cyclooxygenase 2 (COX2) was determined. The dose of protein hydrolysate was selected based on previous studies [24]. Cells were lysed using boiling Laemmli buffer containing 50 Mm dithiothreitol (DTT) and 0.2% Triton-X-100. Cell lysates were loaded onto a 9% separating gel and transferred to a nitrocellulose membrane (diameter 0.45 µm, 1620115, Bio-Rad, Montreal, QC, Canada) for incubation with antibodies. Protein bands of VCAM-1 (ab134047, Abcam, ON, Canada), COX2 (ab15191, Abcam) were normalized against glyceraldehyde 3-phosphate dehydrogenase (GAPDH, ab8245, Abcam). Donkey-anti-rabbit 800 CW or donkey-anti-mouse IRDye 680 RD secondary antibodies (Licor Biosciences, Lincoln, NE, USA) were used to visualize the fluorescent protein bands in a Licor Odyssey BioImager, and quantification of protein bands was performed using Image Studio Lite 5.2 (Licor Biosciences, Lincoln, NE, USA).

### 2.10. Statistical Analysis

Purification and biochemical properties of *S. simulans* QB7 protease were performed in triplicate, and data were analyzed by one-way ANOVA, followed by Dunnett’s multiple tests SPSS software version 21 (SPSS Inc, Chicago, MI, USA). Cellular experiment data were collected from six independent measurements and were analyzed by one-way ANOVA followed by Dunnett’s multiple tests using GraphPad Prism version 9 (San Diego, CA, USA). The significance level (*p* value) was set as 5%.

## 3. Results and Discussion

### 3.1. Effect of Fermentation Factors on Crude Protease Production by S. simulans QB7

**Figure 1:**
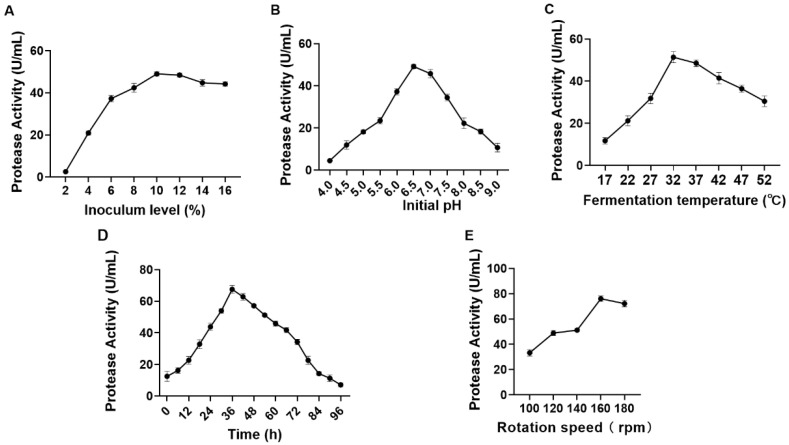
Effects of inoculum ratio (**A**), initial pH (**B**), inoculum time (**C**), inoculum temperature (**D**), and rotation speed (**E**) on the activity of the protease produced by the strain *Staphylococcus simulans* QB7.

The optimal conditions for the production of microbial proteases differed greatly among microorganisms [2]. Figure 1A shows the effect of inoculum ratio on crude protease activity. Crude protease activity gradually increased with an inoculum ratio of 2–10% and then stabilized onwards until 16%. The maximum protease activity (48.96 U/mL) was observed at an inoculum ratio of 10%. Figure 1B shows the effect of the initial pH of the medium on crude protease activity. Protease activity increased within the pH range of 4–6.5 and then decreased steadily until pH 9. Protease activity peaked (49.17 U/mL) at pH 6.5, being nearly five times the activity (11.10 U/mL) of that at pH 9.0. The initial pH value affected the transport of various growth factors regulating microbial metabolism, thereby negatively impacting the production of proteases [28]. These results concurred with previous findings that the initial pH of the medium significantly affected microbial protease production [29,30].

Temperature is one of the key factors regulating microbial growth and metabolism [31], and thus the expression and activity of microbial proteases [32]. The effect of temperature on protease production by *S. simulans* QB7 was investigated. As shown in Figure 1C, the activity of the protease produced by *S. simulans* QB7 increased with the incubation temperature within the range of 17–32 °C and decreased afterward; the highest protease production was reached at the incubation temperature of 32 °C (51.39 U/mL). The optimum incubation temperature for protease production varies widely within the range of 20–50 °C [30]. Crude protease activity gradually increased with an incubation time of up to 36 h (67.57 U/mL), after which it decreased progressively; the activity even went lower than the initial activity after 84 h (Figure 1D). This phenomenon was possibly owing to the sufficiency of the nutrients required for bacterial growth during the early incubation period. As incubation progressed, the nutrients were depleted, and bacterial growth and protease production were restricted. Figure 1E depicts changes in crude protease activity based on the rotation speed. Crude protease activity correlated positively with the rotation speed within 100–160 rpm and was reduced slightly when further increasing the speed to 180 rpm (72.21–76.07 U/mL).

To summarize, the optimal conditions for *S. simulans* QB7 protease production were as follows: inoculum level of 10%, initial pH of 6.5, fermentation temperature of 32 °C, rotation speed of 160, and fermentation time of 36 h.

### 3.2. Protease Purification 

**Figure 2:**
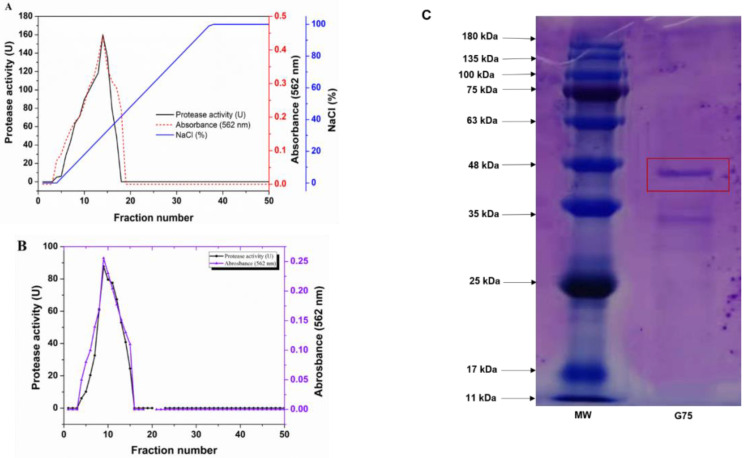
Purification of *Staphylococcus simulans* QB7 protease using DEAE-Sepharose FF column (**A**) and Sephadex-G75 chromatographic column (**B**). SDS-PAGE analysis of the purified *S. simulans* QB7 protease (**C**). MW, protein molecular weight marker; G75, sample after purification with the Sephadex-G75 chromatographic column.

*S. simulans* QB7 crude protease extract was prepared under the optimal conditions as above mentioned. To purify the protease, the extract was dissolved in 80% ammonium sulfate saturation, and the precipitate was collected and dissolved, followed by dialysis for salt removal. As shown in Table 1, ammonium sulfate precipitation increased the purity of the protease by 77%, despite a lower yield of 54.31%; its activity increased from 2.19 ± 0.025 U/mg to 3.8 ± 0.0049 U/mg. The dialyzed protease solution was further purified using DEAE-Sepharose™ FF column (Figure 2A). The results showed that higher protease activity was found for fractions 10–15 than the other fraction, which was washed out using 0.35 M NaCl 0.05 M Tris-HCl buffer (pH 9.0), after which the purity increased by five times, while the yield was further reduced to 32.23%; protease activity was 24.15 ± 0.41 U/mg. Based on the results of the protease activity from the DEAE purification, the 10–15 fractions were combined and loaded onto a gel filtration column (Figure 2B). Fractions 8–13 exhibited higher protease activities than the others. The further purification increased the protease activity to 66.33 U/mg and the yield to 14.75%, respectively (Table 1). Fractions 8–13 were pooled for analysis of protease biochemical characteristics. In addition, the purity and molecular mass of purified protease were determined by SDS-PAGE. After the final purification step, two protein bands were obtained in the pooled active fraction (Figure 2C), whose molecular mass was between 33–47 kDa. These results were consistent with a previous study by Worsztynowicz et al. [33], who reported 18.5–25k Da extracellular protease from *Enterococcus faecalis*. Other researchers reported that extracellular protease with molecular weights of 21.5, 24.0, and 20.0 kDa from *Staphylococcus xylosus* A2 and *Staphylococcus epidermidis* BP9. [13,14,22]. Meanwhile, Wang et al. [12] reported a neutral extracellular protease from *Staphylococcus carnosus* RT6.

### 3.3. Effect of pH and Temperature on Protease Activity and Stability

**Figure 3:**
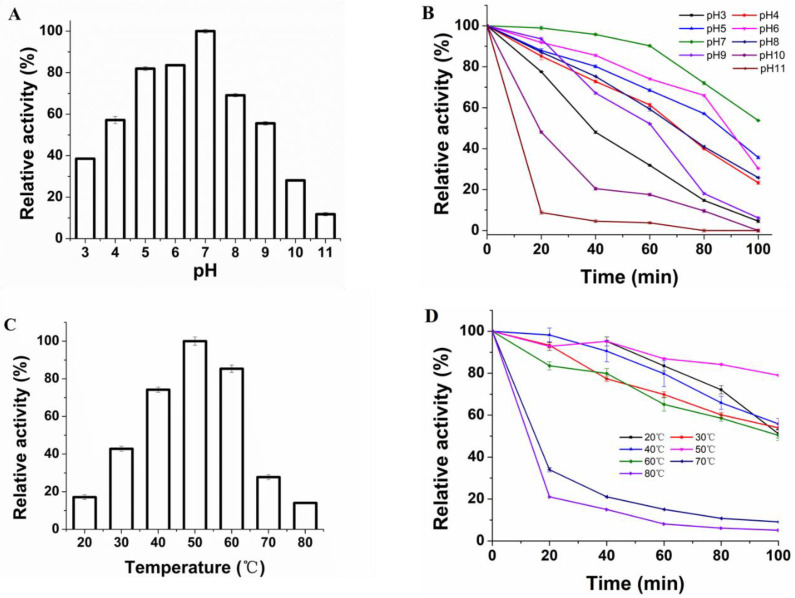
Effects of pH (**A**,**B**) and temperature (**C**,**D**) on the activity and stability of the protease produced by *Staphylococcus simulans* QB7.

The effect of pH (3–11) on the activity of the protease produced by *S. simulans* QB7 was investigated. As shown in Figure 3A, *S. simulans* QB7 protease reached the highest activity at pH 7.0 (the value was defined as 100% of the protease activity). Based on that, the activity of *S. simulans* QB7 protease (expressed as the relative activity at pH 7.0) increased within the pH range of 3.0–7.0 and decreased at pH 11.0. Typically, proteases in fermented meat products often maintained a high activity at a wide range of pH, particularly weakly acidic pHs [9]. Salihi et al. [9] reported alkaline proteases from *aspergillus oryzae* CH93, which had its highest activity at pH 8.0.

Under pH 3–11, the protease stability showed a decreasing trend with time (Figure 3B). After 60 min of incubation, the protease could maintain >50% of activity at pH 4.0–9.0. Its protease was more efficient at a weakly acidic pH (pH 6.0) than at a weakly alkaline pH (pH 8.0). However, *S. simulans* QB7 protease activity was significantly reduced to 31.86%, 17.59% and 3.81%, at pH 3, 10, and 11.0, respectively. These results indicated that *S. simulans* QB7 protease was stable within the pH range from 3.0 to 9.0. Bisswanger [34] reported that the pH could affect the three-dimensional protein structure of the enzyme and the protonation of side amino acids. These results demonstrated that the *S. simulans* QB7 protease exhibited higher activity in the neutral pH environment, which made *S. simulans* QB7 protease suitable for application in weak acid fermentation environments and hydrolysis of meat proteins to bioactive peptides and amino acids.

Figure 3C illustrates the effect of temperature on *S. simulans* QB7 protease activity. It was observed that its activity increased with temperature within the range of 20–50 °C; the activity decreased if the temperature was further increased, especially over 70 °C, at which point the protease almost lost more than 75% of its activity. It has been reported that the three-dimensional structure of enzymes is thermo-sensitive and can be easily destroyed at high temperatures due to denaturation [20,34]. Similar behavior of optimum temperature at 50 °C was observed in other microbial proteases [12,13,22]. The stability of *S. simulans* QB7 protease with time is shown in Figure 3D. The protease activity was decreased at prolonged incubation time at all temperatures. At the temperature of 20–60 °C, the protease could maintain more than 50% of its activity after 100 min of incubation. At higher temperatures (> 70 °C), however, its activity reduced drastically to be below 40% after 20 min of incubation and almost zero after 60 min. These results indicated that *S. simulans* QB7 protease was stable within the temperature range of 20–60 °C. To sum up, the optimal pH of the protease was 7.0, and the temperature was 50 °C. Besides, the protease remained stable at the pH of 3.0–9.0 and temperature of 20–60 °C. Similar results were founded by Sun et al. [29], who reported the similar pHs (3.0–9.0) and temperatures (25–45 °C) of LAB extracellular proteases isolated from Harbin dry sausages, as well as Wang et al. [12,13], who found that the purified extracellular protease produced by CNS isolated from dry sausage was stable at pH 4.0 to 9.0 and temperature 20 to 50 °C. Lücke [35] reported a temperature change from 25 to 27 °C and a pH change from 5.5 to 6.5 during the fermentation process of meat.

### 3.4. Effect of Metal Ions and Inhibitors on the Activity of S. simulans QB7 Protease

**Figure 4:**
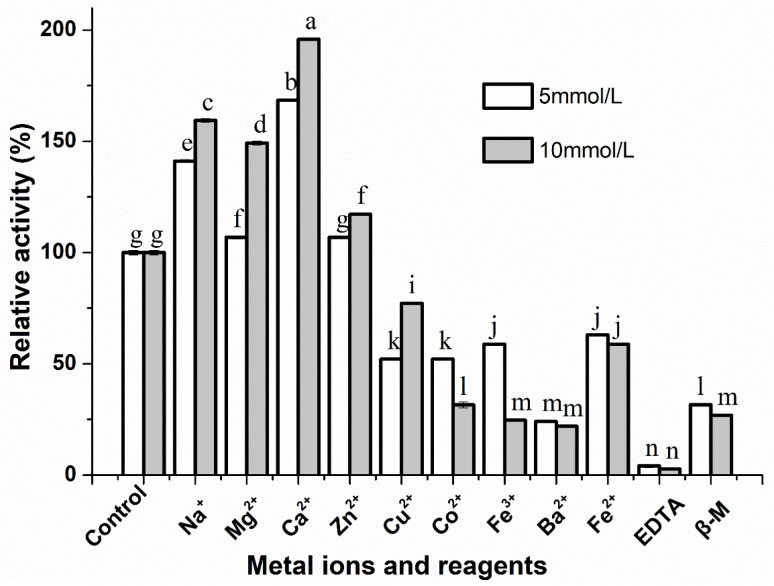
Effects of metallic ions and inhibitors on the activity of the protease produced by *Staphylococcus simulans* QB7. Data are presented as mean ± SD. Different lowercase letters (a–e) indicate significant differences (*p* < 0.05). (Add a space to the legends between 5 and mmol/L.

The effect of metal ions on *S. simulans* QB7 protease activity was evaluated because metal ions constitute the active site of proteases. The group without metal ions was used as the control. As shown in Figure 4, metal ions (Na^+^, Mg^2+^, Ca^2+^, and Zn^2+^) significantly increased the enzymatic activity of *S. simulans* QB7 protease at two different concentrations (5 and 10 mmol/L) (*p* < 0.05). The highest activity was achieved for Ca^2+^. Specifically, Ca^2+^ increased protease activity by 168.49% and 195.89% at the concentration of 5 mmol/L or 10 mmol/L, respectively, However, the protease activity was significantly inhibited (*p* < 0.05) in the presence of Cu^2+^, Co^2+^, Fe^2+^, Ba^2+^, and Fe^3+^. For example, the activity decreased to 24.11% and 21.92% in the presence of Ba^2+^ at 5 mmol/L and 10 mmol/L, respectively. Similar results were reported by Wang et al. [22] and Abdel-Naby et al. [36], who found that relative activities of protease produced by microorganism were strongly enhanced to 157.0 % and 134.3% by Ca^2+^. These metal ions might bind to the protease promoting the internal interaction of protein molecules to improve the enzyme activity [37]. In addition, the effect of protease inhibitors, including β-M and EDTA on protease activity, was investigated. Both EDTA and β-M significantly reduced protease activity at the concentration of 5 and 10 mmol/L (*p* < 0.05). Salihi et al. [9] demonstrated that the relative activities of protease from microorganisms were inhibited by EDTA and β-M. It has been reported that EDTA inhibited protease activity because it could chelate the metal ion, while β-M may reduce the disulfide bond of protease and then affect the protease activity [37].

### 3.5. The Effect of S. simulans QB7 Protease on Protein Degradation and Generating Bioactive Peptides from Meat Proteins

Hydrolysis of meat proteins is considered an important biochemical reaction during fermentation and ripening, which determines the characteristic taste and flavor of fermented meat products [16]. In the present work, the ability of the purified *S. simulans QB7* protease in degrading meat proteins and generating small peptides was assessed; the antioxidant and anti-inflammatory activities of the generated peptides were also evaluated.

#### 3.5.1. Molecular Weight Distribution

**Figure 5:**
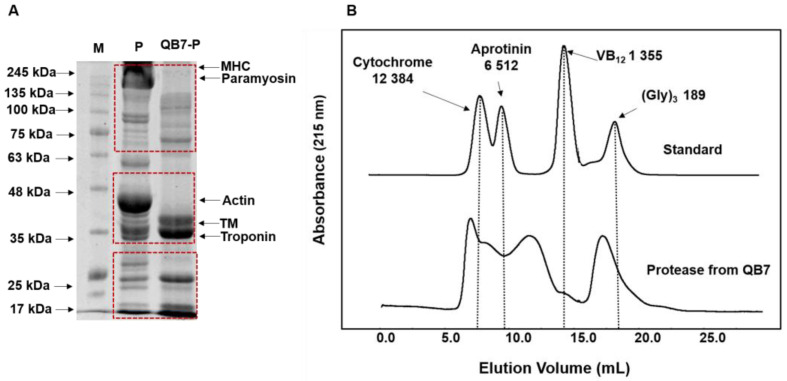
SDS-PAGE (**A**) and size exclusion chromatogram (**B**) of hydrolyzed meat protein prepared by *S. simulans* QB7 protease. M: molecular weight of the protein standard; MP: meat protein; QB7-P: *S. simulans* QB7 protease; MHC: myosin heavy chain; TM: tropomyosin.

SDS-PAGE results of the hydrolyzed meat proteins using *S. simulans* QB7 protease are shown in Figure 5A. There were five major bands in the raw meat proteins, including myosin heavy chain (MHC), paramyosin, actin, tropomyosin (TM), and troponin. MHC, paramyosin, and actin bands disappeared due to the action of *S. simulans* QB7 protease, while the intensity of TM and troponin bands increased. In addition, several major degradation products, with a molecular weight of 100–135 kDa, 63–75 kDa, and 17–25 kDa, were observed. These results were founded to be consistent with earlier studies [13,22], reporting that *S. epidermidis* BP9 protease and *S. xylosus* A2 protease could accelerate the degradation of meat protein and also promote the production of new protein products. These results indicated that the proteases produced by starter cultures have an important role in the degradation of fermented meat products.

Size exclusion chromatography was used to further study the profile of low-molecular-weight peptides (Figure 5B). *S. simulans* QB7 protease had a high efficiency in generating low-molecular-weight peptides from meat proteins, reflecting the large proportion of peptides with molecular weight lower than 3 kDa. A previous study indicated that protease derived from microbial can hydrolyze meat protein into small proteins, such as peptides and amino acids [13]. Moreover, Broncano et al. [38] demonstrated that the peptide fractions <3 kDa are easier to be absorbed and have stronger bioactivity than the large-size peptide fractions. These results indicated that *S. simulans* QB7 proteases could degrade meat proteins and generate small peptides.

#### 3.5.2. Cell Toxicity Assay

**Figure 6:**
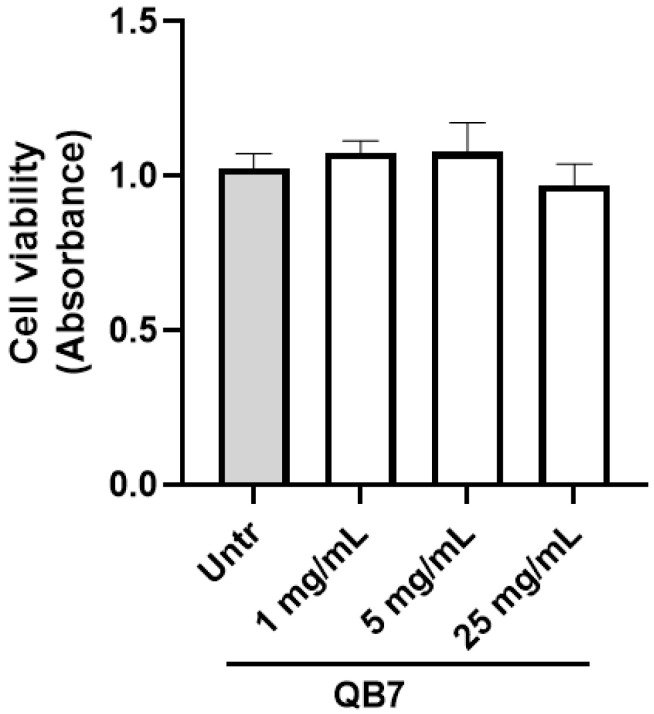
Effect of meat protein hydrolysates on the viability of EA.hy926 cells. Cells were treated with the hydrolysate at the concentrations of 1, 5, and 25 mg/mL for 24 h before subjecting to the alamarBlue^®^ cell viability assay; ns, not significant (*p* > 0.05). Untreated, Untr.

Prior to evaluating the bioactivity of the meat protein hydrolysate, its effect on cell viability was studied, as shown in Figure 6. The viability of treated EA.hy926 cells was slightly increased at the concentrations of 1 mg/mL and 5 mg/mL, which may be due to the fact that the hydrolysate prepared using *S. simulans* QB7 protease provided nutrients that are favorable for cell growth. These results demonstrated that the hydrolysate showed no cytotoxic against EA.hy926 cells at the concentration of up to 5 mg/mL over 24 h (*p* > 0.05). Based on previous studies, we selected the concentration of the hydrolysate at 2.5 mg/mL for evaluating its bioactivity in EA.hy926 cells for the subsequent experiments [39,40].

#### 3.5.3. Antioxidant Activity

**Figure 7:**
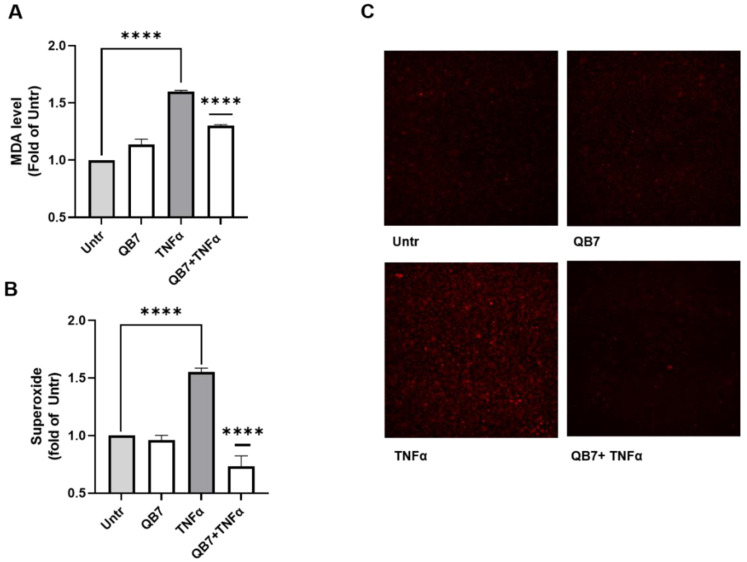
Antioxidant activity of meat protein hydrolysates in TNFα-stressed EA.hy926 cells. Cells were treated with protease hydrolysate (2.5 mg/mL) for 1 h before stimulation with TNFα (10 ng/mL) for 6 h, and malondialdehyde (MDA) levels (**A**) and superoxide production (**B**) were evaluated. A representative set of images are shown (C). Data are expressed as mean ± SEM and normalized against the untreated group (Untr). **** *p* < 0.0001.

The antioxidant activity of meat protein hydrolysate prepared using *S. simulans* QB7 protease was evaluated in human vascular EA.hy926 cells, which are well established cellular models for evaluating health-promoting effects of various bioactive substances, including endothelial dysfunction, inflammation, and oxidative stress [25,41]. In this study, the effect of hydrolysate on oxidative stress was evaluated. Oxidative stress induces various types of damage to cells and disrupts cellular function [42,43]. Aberrant and sustained oxidative stress responses are involved in vascular dysfunction, thus contributing to the incidence of vascular remodeling, hypertension, type 2 diabetes, atherosclerosis, and other cardiovascular diseases [44,45]. Besides, amelioration of oxidative stress has been considered a key preventive strategy for combating various chronic diseases [46,47]. Figure 7A shows the effect of *S. simulans* QB7 protease-digested meat protein hydrolysate on MDA generation in TNFα-stimulated EA.hy926 cells. TNFα stimulation significantly increased MDA level (*p* < 0.0001), which was significantly inhibited by the hydrolysate treatment (*p* < 0.0001). Similarly, the hydrolysate treatment also significantly lowered the superoxide level in TNFα-stimulated EA.hy926 cells (Figure 7B,C). Previous studies demonstrated that EA.hy926 cells are a good model for assessing the effects of bioactive peptides or hydrolysates on endothelial oxidative stress [26,41]. Wang et. al. [13] found that the addition of *S. carnosus protease* increased the concentration of peptides in Harbin dry sausage, while the activities of peptides have not been studied. Yu et al. [15] suggested that *Lactobacillus plantarum* and *Staphylococcus simulans* strains-inoculated meat proteins promoted the formation of antioxidant peptides. In recent years, many antioxidant peptides were founded in fermented meat or meat byproducts, such as dry sausages, dry-cured hams, bone, blood, and skin [10,48]. However, most previously reported antioxidant peptides were based on in vitro biochemical assays, such as DPPH radical scavenging activity, ABTS radical scavenging activity, ferric-reducing antioxidant power assay, and lacked biological relevance [49]. In the present work, the significantly inhibited TNFα-stimulated superoxide and MDA levels (*p* < 0.0001) indicated that *S. simulans* QB7 protease was capable to generating peptides with antioxidant activity from meat proteins.

#### 3.5.4. Anti-Inflammatory Activity

Amelioration of endothelial inflammation is considered one of the important targets for treating hypertension and cardiovascular diseases, hence the protective effect of the hydrolysate on inflammation in EA.hy926 cells was investigated. VCAM-1 and COX2 are two inflammatory mediators in endothelial cells [25,32,50], thus the expression of these two proteins in EA.hy926 cells was used to evaluate the anti-inflammatory activity of *S. simulans* QB7 protease-digested meat protein hydrolysate in the present study.

**Figure 8:**
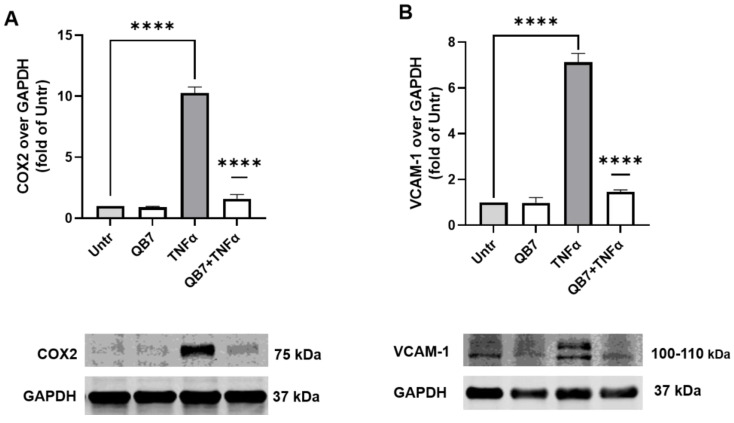
Effect of protease hydrolysate treatment on TNFα-induced inflammation in EA.hy926 cells. Cells were treated with protease hydrolysates (2.5 mg/mL) for 24 h before TNFα (10 ng/mL) stimulation for another 6 h. Subsequently, cell lysates were used to determine the protein expression of COX2 (**A**) and VCAM-1 (**B**) using Western blotting. Protein bands were normalized to GAPDH expression. Data were expressed as mean ± SEM and normalized to the untreated group (Untr). **** *p* < 0.0001.

As shown in Figure 8, VCAM-1 and COX2 levels surged in EA.hy926 cells upon TNFα stimulation (*p* < 0.0001), whereas the hydrolysate treatment significantly inhibited both levels (*p* < 0.0001). These results suggested that *S. simulans* QB7 protease-digested meat protein hydrolysate has a better effect on inhibition the level of the inflammatory protein than chicken muscle protein-derived peptide VVHPKESF in EA.hy926 cells [25,27,39]. In the previous studies, researchers demonstrated that meat protein hydrolysate prepared using protease generated by CNS, including *Staphylococcus carnosus*, *Staphylococcus xylosus*, and *Staphylococcus epidermidis*, increased the concentration of peptide [13,14,22]. The activities of generated peptides have not been evaluated. In the present study, the anti-inflammatory activity of the hydrolysate produced by *S. simulans* QB7 protease was evaluated. These findings suggested the formation of anti-inflammatory peptides by the *S. simulans QB7* protease from meat protein. In recent years, researchers have been increasingly interested in anti-inflammatory peptides derived from fermented meat products, as well as the effect of added starter cultures on the formation of peptides during fermentation and ripening [16,48]. The present study indicates a promising approach, using adding starter cultures to produce anti-inflammatory peptides in fermented meat products.

## 4. Conclusions

In the present study, the extracellular protease produced by *S. simulans* QB7 was purified, and the properties of the purified protease were investigated. *S. simulans* QB7 protease showed high capacity in hydrolyzing meat proteins, and optimal conditions for crude enzymatic activity were as follows: inoculum level of 10%, initial pH of 6.5, fermentation temperature of 32 °C, rotation speed of 160, and fermentation time of 36 h. In addition, in the presence of Na^+^, Mg^2+^, Ca^2+^, and Zn^2+^, protease activity was significantly (*p* < 0.05) activated, whereas the activity was inhibited in the presence of Cu^2+^, Co^2+^, Fe^3+^, Ba^2+^, Fe^2+^, β-M, and EDTA. Furthermore, the protease was able to degrade meat proteins effectively, and the obtained hydrolysate exhibited great antioxidant and anti-inflammatory activities in EA.hy926 cells. Taken together, this study reported the enzymatic properties of *S. simulans* QB7 protease, its efficiency in hydrolyzing meat proteins, and the health-beneficial effects of obtained peptides, which could provide the basis for its future applications in fermented meat products.

## Figures and Tables

**Table 1:** Characteristics of the protease produced by *Staphylococcus simulans* QB7 after purification.

Purification Steps	Total Activity (U)	Total Protein (mg)	Enzymatic Activity (U/mg Protein)	Purificatio Fold	Yield (%)
Culture medium supernatant	107.90 ± 1.22	49.38 ± 0.021	2.19 ± 0.025	1	100
Ammonium sulfate precipitation	58.60 ± 1.23	15.10 ± 0.011	3.88 ± 0.049	1.77	54.31
DEAE-Sepharose FF	34.78 ± 1.61	1.44 ± 0.012	24.15 ± 0.41	15.88	32.23
Sephadex-G75 chromatography	15.92 ± 1.32	0.24 ± 0.077	66.33 ± 1.63	30.29	14.75

Specific activity = total activity (U)/total protein (mg); yield (%) = total activity/total activity of culture medium supernatant × 100; fold purification = specific activity/specific activity of crude extract.

## Data Availability

The data presented in this study are available upon request.

## References

[1] Tavano L., Berenguer-Murcia A., Secundo F. (2018). Biotechnological applications of proteases in food technology. Compr. Rev. Food Sci. Food Saf..

[2] Dos Santos Aguilar H., Sato G. (2018). Microbial proteases: Production and application in obtaining protein hydrolysates. Food Res. Int..

[3] Yu P., Huang X., Ren Q., Wang X. (2019). Purification and characterization of a H_2_O_2_-tolerant alkaline protease from *Bacillus* sp. ZJ1502, a newly isolated strain from fermented bean curd. Food Chem..

[4] Naveed M., Nadeem F., Mehmood T., Muhammad Bilal M., Anwar Z., Fazeeha Amjad F. (2021). Protease—A Versatile and Ecofriendly Biocatalyst with Multi-Industrial Applications: An Updated Review. Catal. Lett..

[5] Ahangari H., Yazdani P., Ebrahimi V., Soofiyani R., Azargun R., Tarhriz V., Eyvazi S. (2021). An Updated review on production of food derived bioactive peptides; focus on the psychrotrophic bacterial proteases. Biocatal. Agric. Biotechnol..

[6] Razzaq A., Shamsi S., Ali A., Ali Q., Sajjad M., Malik A., Ashraf M. (2019). Microbial proteases applications. Front. Bioeng. Biotechnol..

[7] Mohammad Badrud Duza M. (2013). Microbial enzymes and their applications-a review. Indo Am. J. Pharm. Res..

[8] Ullah N., Rehman M.U., Sarwar A., Nadeem M., Nelofer R., Shakir H.A., Irfan M., Idrees M., Naz S., Nabi G. (2022). Purification, Characterization, and Application of Alkaline Protease Enzyme from a Locally Isolated Bacillus cereus Strain. Fermentation.

[9] Salihi A., Asoodeh A., Aliabadian M. (2017). Production and biochemical characterization of an alkaline protease from *Aspergillus oryza*e CH93. Int. J. Biol. Macromol..

[10] Toldrá F., Gallego M., Reig M., Aristoy M., Mora L. (2020). Recent Progress in Enzymatic Release of Peptides in Foods of Animal Origin and Assessment of Bioactivity. J. Agric. Food Chem..

[11] Candogan K., Wardlaw B., Acton C. (2009). Effect of starter culture on proteolytic changes during processing of fermented beef sausages. Food Chem..

[12] Wang H., Wang Q., Xia X., Sun F., Kong B. (2021). Biochemical properties of extracellular protease from *Staphylococcus carnosus* RT6 isolated from Harbin dry sausages, and its hydrolysis of meat proteins. J. Food Sci..

[13] Wang H., Xu J., Kong B., Liu Q., Xia X., Sun F. (2022). Purification and Characterization of the Protease from *Staphylococcus xylosus* A2 Isolated from Harbin Dry Sausages. Foods.

[14] Kong Y., Feng M., Sun J. (2020). Effects of *Lactobacillus plantarum* CD101 and *Staphylococcus simulans* NJ201 on proteolytic changes and bioactivities (antioxidant and antihypertensive activities) in fermented pork sausage. LWT-Food Sci. Technol..

[15] Yu D., Feng M., Sun J., Xu X., Zhou G. (2020). Protein degradation and peptide formation with antioxidant activity in pork protein extracts inoculated with *Lactobacillus plantarum* and *Staphylococcus simulans*. Meat Sci..

[16] Yu D., Feng M., Sun J. (2021). Influence of mixed starters on the degradation of proteins and the formation of peptides with antioxidant activities in dry fermented sausages. Food Control.

[17] Li H., Zhu Q., Chen X., Zhou J., Wu J. (2022). Isolation and characterization of coagulase negative *staphylococci* with high proteolytic activity from dry fermented sausages as a potential starter culture. Food Res. Int..

[18] Farhadian S., Asoodeh A., Lagzian M. (2015). Purification, biochemical characterization, and structural modeling of a potential htrA-like serine protease from *Bacillus subtilis* DR8806. J. Mol. Catal. B: Enzym..

[19] Liu Z., Wang Z., Zhang J. (2008). An acidic protease from the grass carp intestine (*Ctenopharyngodon idellus*). Comp. Biochem. Physiol. Part B Biochem. Mol. Biol..

[20] Afsharnezhad M., Shahangian S., Sariri R. (2019). A novel milk-clotting cysteine protease from *Ficus johannis*: Purification and characterization. Int. J. Biol. Macromol..

[21] Laemmli K. (1970). Cleavage of Structural Proteins during the Assembly of the Head of Bacteriophage T4. Nature.

[22] Wang H., Liu J., Chen Q., Kong B., Sun F. (2021). Biochemical properties of extracellular protease from *Staphylococcus epidermidis* isolated from Harbin dry sausages and its hydrolysis of meat protein. Food Biosci..

[23] Wang C., Wu J. (2012). Preparation and characterization of adhesive from spent hen proteins. Int. J. Adhes. Adhes..

[24] Fan H., Yu W., Liao W., Wu J. (2020). Spent Hen Protein Hydrolysate with Good Gastrointestinal Stability and Permeability in Caco-2 Cells Shows Antihypertensive Activity in SHR. Foods.

[25] Fan H., Bhullar S., Wang Z., Wu J. (2022). Chicken Muscle Protein-Derived Peptide VVHPKESF Reduces TNFα-Induced Inflammation and Oxidative Stress by Suppressing TNFR1 Signaling in Human Vascular Endothelial Cells. Mol. Nutr. Food Res..

[26] Fan H., Wang J., Liao W., Jiang X., Wu J. (2019). Identification and Characterization of Gastrointestinal-Resistant Angiotensin-Converting Enzyme Inhibitory Peptides from Egg White Proteins. J. Agric. Food Chem..

[27] Fan H., Bhullar K., Wu J. (2021). Spent Hen Muscle Protein-Derived RAS Regulating Peptides Show Antioxidant Activity in Vascular Cells. Antioxidants.

[28] Singh S., Tripathi V., Jain R., Vikram S., Garg S. (2010). An antibiotic, heavy metal resistant and halotolerant Bacillus cereus SIU1 and its thermoalkaline protease. Microb. Cell Factories.

[29] Sun F., Sun Q., Zhang H., Kong B., Xia X. (2019). Purification and biochemical characteristics of the microbial extracellular protease from *Lactobacillus curvatus* isolated from Harbin dry sausages. Int. J. Biol. Macromol..

[30] Sharma K., Kumar R., Kumar A. (2017). Microbial alkaline proteases: Optimization of production parameters and their properties. J. Genet. Eng. Biotechnol..

[31] Gupta A., Khare S. (2007). Enhanced production and characterization of a solvent stable protease from solvent tolerant *Pseudomonas aeruginosa* PseA. Enzym. Microb. Technol..

[32] Chen Y., Zhang H., Mats L., Liu R., Deng Z., Mine Y., Tsao R. (2019). Anti-inflammatory Effect and Cellular Uptake Mechanism of Peptides from Common Bean (*Phaseolus vulga* L.) Milk and Yogurts in Caco-2 Mono- and Caco-2/EA.hy926 Co-culture Models. J. Agric. Food Chem..

[33] Worsztynowicz P., Bialas W., Grajek W. (2019). Integrated approach for obtaining bioactive peptides from whey proteins hydrolysed using a new proteolytic lactic acid bacteria. Food Chem..

[34] Bisswanger H. (2020). Enzyme assays. Perspect. Sci..

[35] Lücke F. (2016). Fermented Meat Products: An Overview. Fermented Meat Prod. Health Asp..

[36] Abdel-Naby M., Ahmed S., Wehaidy H., El-Mandy S. (2017). Catalytic, kinetic and thermodynamic properties of stabilized *Bacillus stearothermophilus* alkaline protease. Int. J. Biol. Macromol..

[37] Jaouadi N., Rekik H., Ben Elhoul M., Rahem F., Hila C., Ben Aicha C., Badis A., Toumi A., Bejar S., Jaouadi B. (2015). A novel keratinase from *Bacillus tequilensis* strain Q7 with promising potential for the leather bating process. Int. J. Biol. Macromol..

[38] Broncano J., Timon M., Parra V., Andres A., Petron M. (2011). Use of proteases to improve oxidative stability of fermented sausages by increasing low molecular weight compounds with antioxidant activity. Food Res. Int..

[39] Fan H., Liao W., Wu J. (2022). Chicken muscle hydrolysate reduces blood pressure in spontaneously hypertensive rats, upregulates ACE2, and ameliorates vascular inflammation, fibrosis, and oxidative stress. J. Food Sci..

[40] Liang Q., Chalamaiah M., Ren X., Ma H., Wu J. (2018). Identification of New Anti-inflammatory Peptides from Zein Hydrolysate after Simulated Gastrointestinal Digestion and Transport in Caco-2 Cells. J. Agric. Food Chem..

[41] Baranska P., Jerczynska H., Pawlowska Z., Koziolkiewicz W., Cierniewski S. (2005). Expression of Integrins and Adhesive Properties of Human Endothelial Cell Line EA.hy 926. Cancer Genom. Proteom..

[42] Touyz M., Schiffrin L. (2000). Signal transduction mechanisms mediating the physiological and pathophysiological actions of angiotensin II in vascular smooth muscle cells. Pharmacol. Rev..

[43] Touyz M., Schiffrin L. (2004). Reactive oxygen species in vascular biology: Implications in hypertension. Histochem. Cell Biol..

[44] Galle J., Quaschning T., Seibold S., Wanner C. (2003). Endothelial dysfunction and inflammation: What is the link?. Kidney Int..

[45] Odegaard O., Jacobs R., Sanchez A., Goff C., Reiner P., Gross D. (2016). Oxidative stress, inflammation, endothelial dysfunction and incidence of type 2 diabetes. Cardiovasc. Diabetol..

[46] Chen X., Touyz M., Park B., Schiffrin L. (2001). Antioxidant Effects of Vitamins C and E Are Associated with Altered Activation of Vascular NADPH Oxidase and Superoxide Dismutase in Stroke-Prone SHR. Hypertension (Dallas, Tex. 1979).

[47] Fleenor S., Seals R., Zigler L., Sindler L. (2012). Superoxide-lowering therapy with TEMPOL reverses arterial dysfunction with aging in mice. Aging Cell.

[48] Gallego M., Mora M., Hayes M., Reig M., Toldra F. (2019). Peptides with Potential Cardioprotective Effects Derived from Dry-Cured Ham Byproducts. J. Agric. Food Chem..

[49] Jahandideh F., Chakrabarti S., Davidge S., Wu J. (2016). Antioxidant Peptides Identified from Ovotransferrin by the ORAC Method Did Not Show Anti-Inflammatory and Antioxidant Activities in Endothelial Cells. J. Agric. Food Chem..

[50] Yamawaki H., Kuramoto J., Kameshima S., Usui T., Okada M., Hara Y. (2011). Omentin, a novel adipocytokine inhibits TNF-induced vascular inflammation in human endothelial cells. Biochem. Biophys. Res..

